# Epigenetic and Genetic Keys to Fight HPV-Related Cancers

**DOI:** 10.3390/cancers15235583

**Published:** 2023-11-25

**Authors:** Veronica Folliero, Federica Dell’Annunziata, Annalisa Chianese, Maria Vittoria Morone, Francesca Mensitieri, Federica Di Spirito, Antonio Mollo, Massimo Amato, Massimiliano Galdiero, Fabrizio Dal Piaz, Pasquale Pagliano, Luca Rinaldi, Gianluigi Franci

**Affiliations:** 1 Department of Medicine, Surgery and Dentistry “Scuola Medica Salernitana”, University of Salerno, 84081 Baronissi, Italy; vfolliero@unisa.it (V.F.); federica.dellannunziata@unicampania.it (F.D.); fmensitieri@unisa.it (F.M.); fdispirito@unisa.it (F.D.S.); amollo@unisa.it (A.M.); mamato@unisa.it (M.A.); fdalpiaz@unisa.it (F.D.P.); ppagliano@unisa.it (P.P.); 2 Department of Experimental Medicine, University of Campania “Luigi Vanvitelli”, 80138 Naples, Italy; annalisa.chianese@unicampania.it (A.C.); mariavittoria.morone@unicampania.it (M.V.M.); massimiliano.galdiero@unicampania.it (M.G.); 3 Department of Medicine and Health Sciences “Vincenzo Tiberio”, Università degli Studi del Molise, 86100 Campobasso, Italy

**Keywords:** human papillomavirus, epigenetic regulation, cancer, combinatorial therapies

## Abstract

**Simple Summary:**

Human papillomavirus (HPV) infection is a highly prevalent sexually transmitted disease globally. Although most HPV infections do not result in cancer, certain HPV strains are strongly associated with most cervical cancers, as well as with some cases of anogenital and oropharyngeal cancers. Epigenetic modifications have been revealed to impact the cellular pathways involved in the emergence of the neoplastic phenotype. The inherent reversibility and dynamic nature of epigenetic modifications render enzymes as epigenetic proper targets for the development of effective therapeutic strategies against HPV-induced tumors.

**Abstract:**

Cervical cancer ranks as the fourth most prevalent cancer among women globally, with approximately 600,000 new cases being diagnosed each year. The principal driver of cervical cancer is the human papillomavirus (HPV), where viral oncoproteins E6 and E7 undertake the role of driving its carcinogenic potential. Despite extensive investigative efforts, numerous facets concerning HPV infection, replication, and pathogenesis remain shrouded in uncertainty. The virus operates through a variety of epigenetic mechanisms, and the epigenetic signature of HPV-related tumors is a major bottleneck in our understanding of the disease. Recent investigations have unveiled the capacity of viral oncoproteins to influence epigenetic changes within HPV-related tumors, and conversely, these tumors exert an influence on the surrounding epigenetic landscape. Given the escalating occurrence of HPV-triggered tumors and the deficiency of efficacious treatments, substantial challenges emerge. A promising avenue to address this challenge lies in epigenetic modulators. This review aggregates and dissects potential epigenetic modulators capable of combatting HPV-associated infections and diseases. By delving into these modulators, novel avenues for therapeutic interventions against HPV-linked cancers have come to the fore.

## 1. Introduction

Per data provided by the World Health Organization (WHO), cancers rank among the leading contributors to global mortality, accounting for roughly 10 million deaths in the year 2020 [1]. Viral infections have a hand in approximately 15–20% of all human cancers, underscoring the substantial impact of several viruses on the emergence of malignancies [2]. Among these contributors, the human papillomavirus (HPV) holds significance in the context of cancer-related statistics. HPVs are epitheliotropic viruses infecting squamous epithelia (skin and mucosae) belonging to the family Papillomaviridae [3]. Nearly 200 types of human HPV have been identified and classified into two groups based on their carcinogenic properties: low-risk HPV (LR-HPV) and high-risk (HR-HPV). LR-HPVs mainly include genotypes 1, 2, 4, 6, 11, 40, 42, 43, 44, 61, 70, 72, and 81. They induce hyperproliferative lesions on the hands, feet, and genital and laryngeal areas and seldom advance to high-grade neoplasia and invasive malignancy [4]. Otherwise, HR-HPVs consist of the genotypes 16, 18, 31, 33, 35, 39, 45, 51, 51, 56, 58, 59, 68, 73, and 82. It is currently established and well-documented that HPV16 and 18 are the most potent high-risk genotypes, accounting for roughly 70% of all incidences of invasive cervical cancer across the globe [5]. 

The pathogenesis of HPV-associated tumors is due to the integration of the viral genome into the host cell DNA, leading to the expression of viral E6 and E7 oncoproteins. The latter inactivate the tumor suppressor proteins p53 and pRb, respectively, establishing the neoplastic phenotype [6]. 

Despite the wide availability of vaccines against HR-HPV infection from 2006 to 2008, their administration has been limited mainly to girls due to their prophylactic nature [7]. Furthermore, their uptake has been disappointing in several countries, and alarmingly, some countries with initially high vaccination rates have recently seen steep declines in acceptance [7]. Compounding the problem, these vaccines have the distinction of being the most expensive ever produced. Consequently, their implementation in developing countries, where more than 80% of significant HPV-associated diseases occur, presents considerable challenges [8]. This underscores the need to develop new therapies to complement the global initiative to address HPV-associated diseases. 

Throughout HR-HPV infection, numerous epigenetic alterations have been detected in both the viral and host cell genomes [9]. These encompass instances of viral DNA experiencing hypomethylation or hypermethylation, coupled with hypermethylation of tumor suppressor genes within the host cell. Additionally, changes in histones and modifications in the expression of non-coding RNAs (ncRNAs) have also been observed [10]; for instance, the viral E6 and E7 oncoproteins interact with or complexly modify a range of cellular proteins involved in epigenetic control. These interactions lead to heightened enzymatic activity that drives histone modifications and remodeling of chromatin structure, resulting in a significant change in host gene expression patterns [11]. Moreover, the E2 gene includes CpG islands that are susceptible to methylation. These modifications involve the inhibition of the transcriptional regulatory function of the E6 and E7 oncogenes, leading to their overexpression [12]. Considering the role of epigenetic modifications in the development of the neoplastic phenotype and their reversibility make epigenetic enzymes promising candidates for the development and selection of therapeutic approaches of impact against HPV-induced tumors. Situated within this framework, our review aims to summarize the current available evidence regarding aspects related to HPV infection and related cancers. Furthermore, the epigenetic changes in the carcinogenesis process induced by HR-HPV infections and the epigenetic modulators that could be implicated as potential therapeutic strategies against HPV-induced cancers are addressed. Understanding the molecular mechanisms of HPV-associated cancers and their epigenetic regulation is crucial for the development of personalized therapies and prevention strategies [6,13,14]. 

## 2. Human HPV Features

### 2.1. HPV Structures

**Figure 1:**
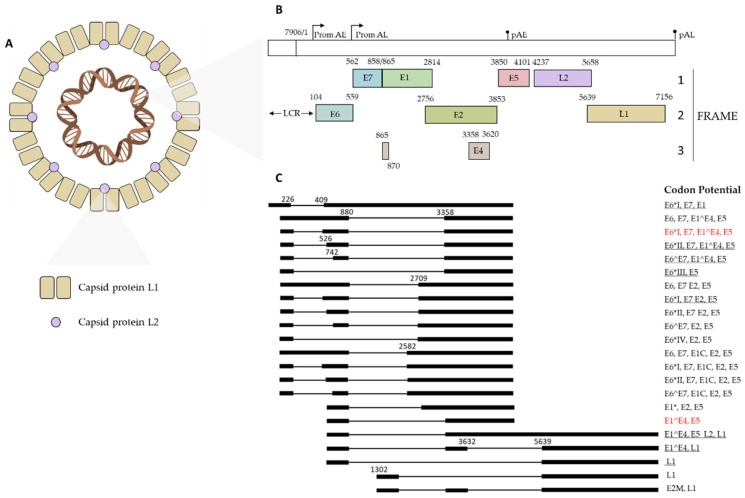
HPV features. (**A**) Structure of the capsid. (**B**) The genome is represented in a linear fashion with the positions of the viral early (PromAE) and late (PromAL) promoters, early (pAE) and late (pAL) polyadenylation signals; below are the predicted viral ORFs with the positions of the nucleotides in the viral genome. (**C**) Coding potentials are illustrated for each transcription event; the most abundant transcripts (>60%) are indicated in red, and the less abundant ones are underlined (<5%).

Human HPVs are non-enveloped viruses belonging to the family Papillomaviridae. They have an icosahedral capsid with a diameter between 50 and 60 nm, inside which contains a circular viral DNA genome associated with histone of 7906 bp [15]. The HPV capsid is composed of two key proteins: the major and minor structural proteins L1 and L2. The outer shell of the virion contains 360 units of the major structural protein L1 arranged in 72 pentameric capsomers. Interactions between these capsomeric units are achieved through the C-terminal section of the L1 protein, and mature viral particles exhibit a stiffer conformation, further stabilized by disulfide bonds. The viral capsid also includes the L2 capsid protein, which is present in variable but smaller amounts. Usually, some of its regions are exposed on the surface of the mature virion, while a significant portion of the protein remains hidden inside (Figure 1A) [16,17,18]. 

The arrangement and function of the HPV genome remain consistent across the Papillomaviridae family, delineated into three primary regions: (i) early region, (ii) late region, and non-coding regulatory region known as the “long control region” (LCR) [19,20]. These three regions are divided by two polyadenylation (pA) sites: early pA (AE) sites and late pAL (AL) sites [20]. Moreover, two viral promoters, categorized as early (p97 for HPV16 and 31, p105 for HPV18) and late (p742 for HPV31, p670 for HPV16, p811 for HPV18), oversee the regulation of viral gene expression and exhibit activity during distinct phases of the life cycle [21]. In detail, the early promoter is located upstream of the E6 ORF and has a TATA box 35–nt upstream of its transcription start site; it drives the transcription of viral early proteins. Instead, the late promoter lacks the TATA box and is responsible for the transcription of late genes [22]. The HPV genome exhibits polycistronic characteristics, employing various forms of alternative splicing to produce the resulting HPV mRNA transcripts [20]. The early region, denoted by “E”, occupies over 50% of the virus genome and encodes six open reading frames (ORFs), such as E1, E2, E6, E7, E4, and E5 [23]. The late gene region, indicated with “L”, covers almost 40% of the virus genome and consists of ORFs L2 and L1 [23]. The latter codes for the structural proteins and the major and minor capsid proteins, respectively. Spanning approximately 10% of the genome, the long control region (LCR) is a non-coding region situated between the termination point of ORF L1 and the initiation codon E6 [24]. LCR contains the origin of replication and recognition sites for host and viral transcription factors [25]. HPV16 exploits three coding frames to translate viral proteins. In frame 1, the genes E7, E1, E5, and L2 are expressed. The genes E6, E2, and L1 exhibit expression in frame 2, whereas exclusive expression in frame 3 is attributed solely to the E4 gene (Figure 1B,C) [26]. 

E1 is a highly conserved protein of approximately 68 kDa and plays a central role in the viral genome replication event. It binds weakly to the origin of replication in LCR; however, this binding is significantly enhanced through the formation of a complex with the corresponding E2 protein [27]. The primary function of E2 is to upload E1 to the replication source. They work together to recruit replication proteins from the host cell to the origin of replication [8]. E1 also possesses intrinsic ATPase and 3′–5′ helicase activities, critical for the unfolding of the origin and progression of replication forks [28].

E2 is a protein of about 50 kDa, required for both replication and transcription processes. It consists of three domains: an N-terminal conserved transactivating domain (TAD), a hinge region, and a DNA binding domain (DBD) [29]. TAD mediates interactions with viral and host proteins. In detail, this domain mediates interactions with the E1 protein to initiate the viral genome replication event [30]. It interacts with the E7 protein, inhibiting its transforming function [31]. The TAD domain of HR-HPV E2 protein can indirectly induce apoptosis by binding the anaphase promoter complex (APC) Cdh1 and cdc20 activators [32]. Furthermore, it binds bromodomain protein 4 (Brd4), promoting the integration of the HPV genome with segregating mitotic chromosomes [33]. Interactions between p53 and the DBD domain of the E2 protein have been highlighted, suggesting that it can directly induce apoptosis [34]. Through this domain, E2 can act as an activator or repressor of the transcription of oncogenes (E6 and E7) depending on its abundance, length (isoforms generated by alternative splicing), and protein factors of the host it binds [35]. Together, these proteins cooperate in the early induction of the DNA damage response (DDR) pathway by contributing to a permissive environment for viral genome replication [36]. 

E4 is a phosphoprotein in the range of 10–20 kDa, characterized by late-phase expression, occurring before the expression of the late genes L1 and L2 [37]. Elevated levels of E4 trigger a cell cycle arrest in the G2 phase [38]. Furthermore, its importance in viral genome replication has been underlined, attributed to its interactions with the E2 protein [39]. E4 is endowed with a leucine-rich cluster near its N terminus, a key factor contributing to self-association at the C terminus, forming fibrous structures. These structures play a fundamental role in orchestrating the arrangement of cytokeratins, essential for the liberation of nascent virions [38]. Its role as a transcriptional inducer is important, promoting the expression of the late L1 and L2 genes [40]. 

The HPV E5 protein is a small hydrophobic protein of about 80aa in length, located at the Golgi apparatus, endoplasmic reticulum, and nuclear membrane in the host cell [41]. It consists of three alpha-helices and unstructured segments that protrude outside the lipid bilayer. It interacts with the epidermal growth factor (EGF) receptor, increasing the cell proliferative activity [42]. Furthermore, E5 allows the evasion of the immune response, allowing the accumulation of MHCI in the Golgi apparatus and consequently reducing the recognition of the virus by CD8 T-cells [43]. 

The E6 and E7 proteins are responsible for inducing and maintaining cellular transformation [44]. 

E6 is an oncoprotein of 150 amino acids containing zinc-binding domains necessary for its activity [45]. It promotes p53 degradation through the ubiquitin-dependent proteolytic pathway through interaction with E6-associated protein (E6AP), which is an E3 ubiquitin ligase. Consequently, cells are compelled to engage in uncontrolled cellular proliferation and circumvent the checkpoints of G1/S and G2/M in the cell cycle [46,47]. 

E7 is a phosphoprotein of approximately 100 amino acids, consisting of three conserved regions designated CR-1, -2, and -3 [48]. This protein mediates interactions with members of the retinoblastoma (Rb) protein family [49]. The Rb family has a notable member called p130, which associates with the DREAM complex (DP, RB-like, E2F, and MuvB) during G0/G1. At this stage, it plays a crucial role in preventing S-phase progression by suppressing the transcription of E2F-regulated genes. The dynamic composition of DREAM is complexly regulated throughout the cell cycle. In G0/G1, it associates with E2F4 and p130, while in S/G2, it associates with the transcription factor B-myb. The p130 protein emerges as a key target for E7, promoting entry into the S phase. Notably, Nor Rashid et al. demonstrated a significant reduction in p130-DREAM levels in HPV16-transformed cervical carcinoma cells (CaSki and SiHa) compared to control cell lines. However, when specific small hairpin RNAs were used to target E7 expression, notable reformation of p130-DREAM occurred, leading to cell cycle arrest [50]. The same research group also highlighted that E7 of HPV-16, HPV-18, and HPV-33 compromised the DREAM protein complexes in the same way [50]. The binding of HPV16 E7 to p130, facilitated by its LXCXE domain, was found to be indispensable for disrupting p130-DREAM and promoting the S phase of the cell cycle. Indeed, LXCXE domain-deficient p130 mutants were resistant to the oncogenic effects of E7 when expressed [51,52]. The DREAM complex interacts with members of the E2F family, which act as transcriptional activators, enabling cell cycle transition into the G1/S and S phases [53,54]. In uninfected cells during the G1 phase, the DREAM complex forms a close association with E2F, inhibiting the expression of genes involved in cell cycle progression into the S phase. As the cell prepares for division, there is a dynamic change in interactions within the DREAM complex. The complex dissociates from E2F, allowing E2F to become transcriptionally active. This activation of transcription by E2F is a crucial step that causes cells to enter the S phase of the cell cycle [55]. E7 alters the DREAM complex, preventing suppression of the EF2 factor. This results in an unscheduled reentry of cells into the S phase. The cell phase shift is strongly related to the level of phosphorylation by cyclin-dependent kinases (cdks) [56]. E7 deactivates the suppressive functions of Cdk inhibitors such as p21 and p27, thus avoiding arrests in cell cycle progression [57]. 

The L1 and L2 proteins constitute the viral capsid and are known as the major protein and minor capsid protein, respectively. They are proteins with nuclear localization signal sequences at the C-terminal end whose molecular masses of 55–60 kDa and 64–78 kDa by polyacrylamide gel electrophoresis, respectively [58,59,60]. In the late phase of the viral life cycle, they are synthesized in the cytoplasm, then imported and released into the nucleus of terminally differentiated epithelial cells via nuclear import receptors, where they assemble with the viral genome, generating new virions [58,61,62,63]. Although L1 can form the capsid structure, L2 increases its stability and allows the entry phase [64] (Table 1).

### 2.2. HPV Life Cycle and Treatment Strategies

HPV enters the epithelium through micro-abrasions and hair follicles [8,67]. In cases of HR-HPV infection within the cervical epithelium, entry occurs through the single-layer squamous cell junctions connecting the endocervix and ectocervix [68]. Successful infection requires targeting actively dividing basal or stem epithelial cells [69]. The process involves the interaction of the HPV L1 capsid protein with cell receptors found on the basement membrane or the cell surface within the basal layer. Initial binding primarily relies on heparin sulphate proteoglycans (HSPGs), acting as the primary receptors [70]. Upon binding to HSPGs, a conformational change is triggered in the viral capsid due to cyclophilin B-mediated events. This change exposes the N-terminus of component L2 on the surface of the virion. Furin and/or PC5/6 enzymes subsequently cleave the exposed N-terminus, facilitating the binding of the virus to a secondary receptor located on the target cell membrane [70]. Recent research has unveiled a range of molecules involved in this complex binding process, including EGFR, integrin α6, tetraspanin-enriched membrane microdomains, laminins, syndecan-1, heterotetramer annexin-A2, and vimentin [8,71,72,73,74,75,76,77,78]. It’s noteworthy that the choice of receptor strategy could depend on various factors such as the specific HPV strain, the cell type susceptible to infection, and the context of the infection. In some cases, multiple receptor strategies might be applicable within a single infection [8]. The mechanism by which HPV enters cells bears similarity to endocytosis, exhibiting resemblances to macropinocytosis. The viral episomal genome crosses the cell cytoplasm via a tubulin-mediated pathway, reaching the nucleus through the nuclear pores or following the rupture of the nuclear membrane during mitosis [8]. 

Within the nucleus, L2 localizes viral genomes within PML nuclear bodies, promoting early gene expression. There is evidence that strongly indicates that the RNA responsible for encoding E1 and E2 stands as the earliest detectable RNA species. These two proteins assume a foundational role in facilitating the commencement of genome replication. The rapid expression of the viral transcription factor E2 is justified by its important role as a transcriptional regulator [28,79]. This process is crucial in regulating the precise operations of the viral early promoter, which, in turn, governs the expression of the E6 and E7 regulatory proteins, essential for supporting the continued viability of infected cells [80]. In the initial replication phase of the incoming HPV genome, roughly 50–100 episomal copies are generated within each nucleus [47]. These copies are distributed uniformly among daughter cells by forming attachments to the host cell chromosomes via E2 bound to the viral LCR and chromatin-binding proteins [81]. Cells of this nature are primed for differentiation, preparing to ascend to the upper layers of the epithelium, carrying the viral genomes with them and ensuring that the presence of the virus persists throughout their migration [82]. From in situ hybridization research, it was observed that elevated levels of mRNA coding for E6 and E7 oncoproteins were present in the middle layers of epithelial tissue [83,84]. As one moved towards the upper layers of the epithelium, these levels gradually decreased [85]. These oncoproteins play a crucial role in inhibiting keratinocyte differentiation, thus promoting the proliferation of epithelial cells involved in DNA replication [48]. E4 and E5 are expressed in the middle-upper layers. E4 serves a critical function in mature keratinocytes, facilitating the enhancement of the viral genome amplification process. Additionally, it plays an important role in the latter stages of the virus replication cycle, potentially involved in the restructuring of cytokeratin filaments, rendering the cells more fragile and facilitating the release of viral offspring. Moreover, E4 functions as a transcriptional inducer for late genes [37]. Meanwhile, E5 oversees cell division pathways and acts as a protective shield for the virus against the host’s immune response [86]. Research has revealed that multiple miRNAs possess the capability to regulate the expression of viral genes. For instance, Gunasekharan et al. conducted a study illustrating how miR-145 effectively targets specific sequences within the E1 and E2 regions of HPV31 genes. This precise targeting results in the depletion of essential viral replication factors, ultimately leading to a diminished amplification of the viral genome [87]. 

**Figure 2:**
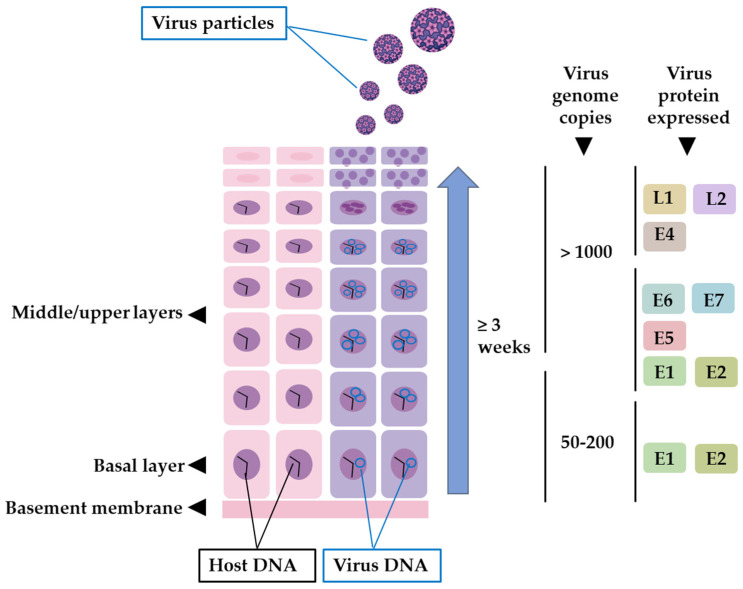
HPV infection, keratinocyte differentiation, and viral protein expression. Pink epithelium layers do not harbor HPVs, while purple host cells are targets for HPV infection.

The late stages of the viral life cycle involve the process of virion formation. This step requires the expression of viral proteins L1 and L2. Interestingly, in the same HPV types (HPV16 and 31), the expression of capsid proteins is delayed until the infected cells reach the granular layer [85]. Several post-transcriptional mechanisms have been elucidated, which potentially contribute to this delay in capsid protein expression. These mechanisms encompass alternative splicing and polyadenylation, mRNA stability, and the regulation of translation [88,89]. L2 synthesis precedes L1 synthesis in differentiated keratinocytes. It is translocated into the nucleus, inducing the rearrangement of nuclear domain 10 (ND10) [83]. Thereafter, L1 is assembled into capsomeres within the cytoplasm. These capsomers are subsequently transported to the nucleus and recruited to ND10, where L2 and capsomeres further assemble into capsids [84]. After their synthesis, viral genomes are known to localize near PML bodies via the E2 protein [90]. As a result, viral genomes are positioned close to viral capsid proteins and prepared for assembly. The interaction between L1 and L2 is critical since L2 probably becomes incorporated into the capsomere core during the viral particle assembly process [20,91,92]. L2 is involved in virion assembly and efficient DNA packaging [60]. Once fully developed, these virions are released into the detaching dead flakes of epithelial tissue. These free virions can persist in the environment for a certain duration and typically reinfect nearby cells, often near the site of their release [68] (Figure 2). 

There are currently no antiviral drugs available that have a direct impact on the life cycle of HPV. The only HPV enzyme on which to direct drug development is E1, which operates as a DNA helicase, allowing the replication of the viral genome. However, efforts to find small molecule inhibitors targeting E1 for use as antivirals have thus far had limited success. Given the absence of other viral enzymes, the focus of antiviral exploration has shifted predominantly towards the modulation of protein-protein interactions [93]. An example is covered by Edwards et al., which showed the inhibitory potential of N-methylpyrrole-imidazole polyamides on HR-HPV replication, resulting in a notable reduction in viral load without inducing cytotoxicity. The compound showed safety up to a concentration of 200 µM. Its mechanism of action involved E1– and E2-mediated termination of HPV replication by precisely targeting their binding sites in the viral origin of replication [94].

### 2.3. HPV Carcinogenesis and Vaccine/Treatment Strategies

**Figure 3:**
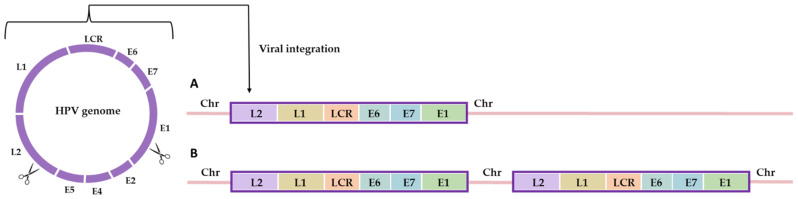
Integration of HPV into human cells: (**A**) a single copy of the viral genome integrates; (**B**) clonal integration event of the viral genome.

Despite the well-established evidence connecting HR-HPVs to the development of various cancers, the likelihood of an HPV infection progressing to HPV-associated cancer remains relatively low [95]. The leading factor contributing to cancer progression appears to be the prolonged infection of basal and stem epithelial cells by at least one HR-HPV over several years [47]. Nevertheless, while persistent infection is a crucial factor, it alone may not be adequate to trigger complete tumorigenesis. In addition to HPV infection, smoking, long-term use of oral contraceptives, and bacterial and other infections have been associated as cofactors for the development of cancer [96]. During the carcinogenesis process, viral DNA integrates into the host genome. Parts of E2 and the adjacent ORFs E4, E5, and part of L1 are routinely lost after integration [97,98]. This genetic alteration results in an increased expression and translation of viral genes, particularly E6 and E7 [80]. For integration to occur, DNA breakpoints are necessary, and these are induced by the presence of HPV E1, E6, and E7 proteins or oxidative stress. Researchers have reported integration hotspots in various genomic regions, including 3q28, 17q21, 13q22.1, 8q24.21, and 4q13.3 [99]. Cells have evolved a specialized mechanism known as the DNA damage response (DDR) to repair DNA damage, whether caused by external factors or internally. Once the DNA damage is successfully repaired, cell cycle checkpoints are resolved, allowing the cell to resume division. However, if the damage is not repaired, the cell can undergo apoptosis [100]. Unremedied DNA breakpoints are a necessary condition for integration to transpire. Viral oncoproteins actively contribute to the maintenance of necessary breakpoints within host chromosomes for integration. Indeed, E6 and E7 disrupt the regulation of cell cycle checkpoints, leading to unprogrammed entry into the S phase and promoting viral genome replication. In this state, the damage response no longer poses an apoptotic threat to the cell, allowing replication to create a host genome with multiple rearranged breakpoints [101]. Proximity between the host and the HPV genome is essential for the successful completion of virus-host fusion. In the HPV life cycle, the HPV genome binds to the host chromatin through the E2-BRD4 complex, facilitating genome partitioning in daughter cells. The association of BRD4 with the integration site suggests that BRD4 might play a crucial role in integration by promoting the binding of viral DNA to the host genome in the DNA damage region [102]. However, the existing literature does not provide a comprehensive explanation for the role of BRD4 in HPV supplementation. To achieve the integration process, the fusion between the virus and the broken double-stranded genome of the host is anticipated through a recombination-directed repair mechanism. Initially, nonhomologous end joining (NHEJ) was involved in HPV supplementation. However, a recent report suggests that the presence of micro-homologous sequences near integration breakpoints indicates the involvement of a homology-mediated DNA repair pathway during the fusion of human and viral DNA. Although sequence homology is observed at the integration site, it is not considered a strict prerequisite [103]. There are two types of integration events: type 1, where a single copy of the viral genome integrates; and type 2, where a concatemer of the viral genome integrates into the host chromosome [104] (Figure 3). 

HPV infections are responsible for approximately 5% of human cancers, with cervical cancer accounting for 99.7% of cases [105]. Most cervical cancers are caused by HR-HPV, particularly HPV-16 [106]. It is estimated that there are over 500,000 new cases and 300,000 deaths related to HPV-associated tumors worldwide each year [107,108]. The prevalence of HPV infections varies depending on the geographic region and the prevalence of different HPV genotypes [107]. To reduce the incidence and mortality of HPV-associated tumors, it is crucial to intensify screening programs and prevention efforts [109]. Cervical screening techniques commonly used include Pap tests, liquid-based cytology (LBC), visual inspection with acetic acid (VIA), and visual inspection with Lugol’s iodine (VILI). Additionally, both prophylactic and therapeutic vaccines are currently being developed. Three prophylactic vaccines against HPV have been approved. The first one, Gardasil, produced by Merck & Co. (Rahway, NJ, USA), was approved in 2006. It is a tetravalent vaccine that contains the L1 proteins of HPV-6, 11, 16, and 18. Gardasil is administered intramuscularly in three doses (at 0, 2, and 6 months) [109]. In 2007, Cervarix, a bivalent vaccine specific for HPV-16 and 18, was approved. It contains L1 proteins of HPV-16 and 18. This bivalent vaccine is also administered intramuscularly in three doses (at 0, 1, and 6 months) [109]. In 2016, a new Gardasil 9-valent vaccine was approved, which protects HPV-6, 11, 18, 31, 33, 45, 52, and 58 genotypes. This vaccine is currently used and administered to males and females aged 9 to 45 years. These three vaccines are recombinant DNA vaccines derived from purified L1 capsid proteins, which can form virus-like particles (VLPs). Only VLPs can induce the production of protective anti-HPV IgG-neutralizing antibodies, preventing the virus from entering host cells [110,111]. Prophylactic vaccines have been shown to protect against the development of high-grade cervical intraepithelial neoplasia (CIN), but they are not effective in treating persistent HPV infections [112,113]. The goals of the therapies for HPV-associated diseases include eradicating infections, alleviating symptoms, and preventing new infections. 

Current therapies have only partially achieved these goals, as most treatments are ablative and do not eliminate the viral infection or transmission. Surgical methods, such as conization, electrocoagulation, and cryotherapy, are commonly used. Drug therapies include retinoids, antivirals, and immunomodulators. Innate and inflammatory response stimulators like Imiquimod are used to treat external genital warts [114]. The mechanism of action of Imiquimod is not entirely clear, but it likely involves binding to the Toll-like Receptor 7 (TLR7) on immune cells, activating helper T lymphocytes. Activated cells secrete cytokines, including alpha interferon [114,115,116,117]. Cidofovir, an antiviral drug primarily used to treat cytomegalovirus retinitis in HIV patients, inhibits viral polymerase, thereby interfering with viral DNA synthesis [118]. Since HPV utilizes the host cell polymerase, Cidofovir is only effective in transformed cells and reduces the oncogenic expression of viral proteins E6 and E7 [119,120,121]. Several lines of evidence have reported that HPV-induced cancer is directly related to numerous epigenetic alterations both in the viral DNA and in the genome of infected cells [10,122]. In this context, epigenetic modulators have gained significant interest as a potential therapy for HPV infections, as discussed in detail in the following paragraph.

## 3. HPV: Epigenetic Modulators

### 3.1. HPV Chromatin Structure and Epigenetic Regulation of Transcription

**Figure 4:**
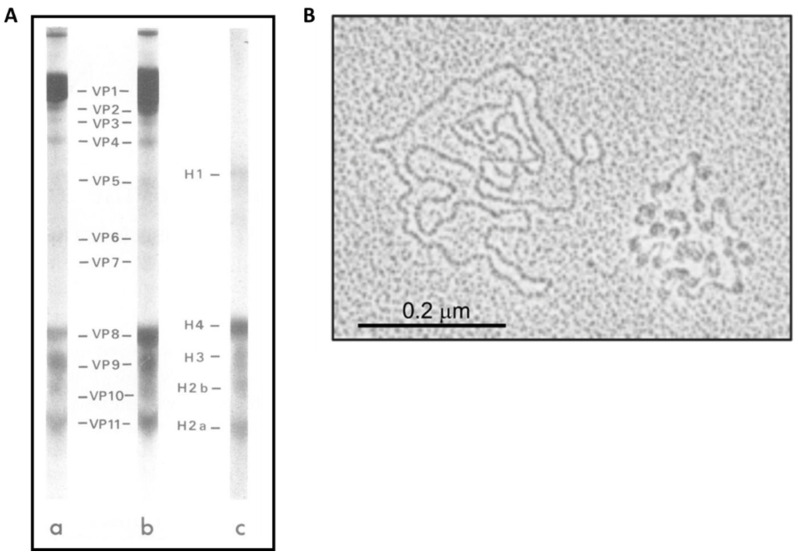
HPV genome organization. (**A**) SDS-polyacrilamide cylindrical gel electrophoresis and gradient slab gel electrophoresis of SDS-dissociated papillomaviruses. Samples included highly purified BPV (a), HPV (b) proteins, and calf liver histones (c); (**B**) electron microscopy of HPV genomes showed naked HPV DNA molecules (left) and nucleoprotein-DNA complexes (right) [116]. (viral protein 1–11, VP 1–11; H1, histone 1; H2A, histone 2A; H2B, histone 2B; H3, histone 3; H4, and histone 4) [125].

The term epigenetics refers to transcriptional regulatory mechanisms that induce heritable changes in the phenotype but not in the genotype [123]. In the virion and infected cells, HPV genomes are structured as nucleosomes compacted into chromatin [124]. The discovery of HPV DNA-associated histone complexes dates to 1977, when Favre and colleagues first reported it. Electrophoretic analysis of highly purified when Favre and colleagues first reported it. Electrophoretic analysis of highly purified viral proteins (VPs) revealed the connection of the viral genome with proteins with molecular masses like those found in the canonical histone complex, namely H1 (histone 1), H2A (histone 2A), H2B (histone 2B), H3 (histone 3), and H4 (histone 4) (Figure 4A). It is estimated that these proteins constitute about 87% of the total proteins associated with DNA. Further examination of the HPV DNA, using electron microscopy, revealed the organization of the viral genome into nucleosomes with a diameter of approximately 12 nm, consistent with the typical nucleosome arrangement. The complete HPV genome showed up to 32 nucleosome complexes, with the interconnecting DNA showing variable lengths, suggesting that the positioning of these nucleosomes depends on specific sequences or regulatory elements within the DNA (Figure 4B) [125]. The placement of nucleosomes on viral enhancer and promoter elements is essential to guide viral transcriptional regulation [126]. Nucleosome mapping studies revealed the presence of at least two nucleosomes within the LCR of HPV 16 and 18. One of these nucleosomes overlaps the viral enhancer, while the second spans the E1 binding site within the origin region of replication and the SP1 binding site in the initial promoter [49]. Strategic positioning of the nucleosome in the early promoter serves to repress virus transcription by preventing the recruitment of SP1. However, the origin of replication and early promoter were found to show weaker affinity for histones than other regions of the viral genome. This suggests that these nucleosomes can be easily rearranged to activate transcription and/or replication [127]. In vitro experiments have shown that an increased concentration of SP1 can displace this nucleosome. Furthermore, the E1 and E2 proteins have been shown to influence nucleosome positioning, indicating that the arrangement of nucleosomes is influenced by both DNA sequence and binding of viral and host factors [128]. In addition, three additional nucleosomes are located along the late promoter, one within the E6 ORF and the others at the 5′ end of the E7 ORF [129]. 

Epigenetic modifications of HPV DNA occurred in the form of methylation of CpG dinucleotides. Sites of methylation were highlighted in the LCR within the E2 binding sites [130]. Post-translational alterations of histones, such as acetylation and methylation, together with DNA methylation, play crucial roles in the epigenetic regulation of HPV genomes [131]. Regulation of viral gene expression in various epithelial layers is essential for the successful progression of the viral life cycle. The achievement of this control mainly depends on the modulation of the viral chromatin structure. HPV has been found to interact with various members of the histone acetyltransferase (HAT) and histone deacetylase (HDAC) families to exert control over viral transcription [132]. Among them, CREB binding protein (CBP) and p300 are notable transcriptional coactivators that interact with transcription factors and acetylated histones. Once CBP/p300 binds to a promoter region, they act as mediators, facilitating the recruitment of factors to trigger transcription [133]. Furthermore, CBP/p300 possess intrinsic HAT activity, which allows them to acetylate histones, causing DNA relaxation within transcriptional promoters, thereby enhancing transcription [134]. CBP/p300-dependent activation of the E6/E7 transcript was observed to be associated with H3 acetylation in the HPV LCR, providing evidence that CBP/p300 affects HPV transcription by altering the epigenetic status of the enhancer/viral promoter [135]. Increased CBP/p300-mediated histone acetylation leads to increased recruitment of the catalytic subunit of the chromatin remodeling complex SWI/SNF, known as Brahma-related gene-1 (Brg1), into the LCR. This Brg1 recruitment is essential for the efficient recruitment of RNA polymerase [136]. Sirtuins (SIRT1-SIRT7), a family of class III HDAC proteins, also participate in the regulation of HPV transcription. In undifferentiated cells, SIRT1 forms complexes with the HPV31 colony-stimulating factor (CSF) and engages in the deacetylation of histone 1 (H1K26Ac) and histone 4 (H4K16Ac) in Lys26 and Lys16, respectively [137]. This dynamic modification of histones by SIRT1 plays a critical role in repressing the transcription of late genes within the HPV genome [138]. However, as cell differentiation progresses, there is a marked change in the interaction between SIRT1 and HPV episomes. The binding affinity of SIRT1 to these episomes undergoes a significant decrease. Consequently, this change in the binding pattern leads to a hyperacetylated state of histone 1, especially Lys26. This hyperacetylation event marks a robust increase in the transcription of these late genes. In undifferentiated keratinocytes, the viral episome adopts a state of chromatin repression primarily due to the presence of repressive histone modifications, including H3K27Me3 and H3K119Ub [10]. However, in differentiated keratinocytes, a shift occurs as viral DNA integrates and the expression of E6 and E7 oncogenes increases. This transition is marked by a more open chromatin configuration within the HPV control region (CSF) and the early promoter. These changes are orchestrated by enzymes like SETD1A and MIL1, which catalyze the deposition of transcriptionally active histone marks, notably H3K4me3. This epigenetic landscape becomes favorable for the recruitment of RNA polymerase II, which, in turn, initiates the transcription of the HPV16 oncogenes from the early promoter [139]. Meanwhile, SETD2, a histone methyltransferase, plays a crucial role in depositing H3K36me3 marks throughout the viral genome. This modification is particularly enriched at the 3′ end of the early gene region, and its presence is vital for both the maintenance and efficient progression of productive viral replication in both undifferentiated and differentiated keratinocytes [140]. The alteration of the methylation of the CpG site plays a fundamental role in the modulation of the transcription of the viral genome under the influence of E2. Indeed, these methylated sites are located within the LCR, effectively preventing the binding of E2 and thus mitigating the repressive impact that E2 exerts on the E6/E7 oncogenes. Investigations of HPV16 episomes within W12 cells, originating from a low-grade cervical lesion, revealed that the viral CSF shows enrichment of methylated CpG dinucleotides in less differentiated cells. However, as these cells undergo differentiation, LCR undergoes a shift towards hypomethylation [141,142].

### 3.2. HPV-Induced Host Epigenetic Changes

Epigenetic modifications are of fundamental importance in the cancer context, facilitating the silencing of tumor suppressor genes, the activation of oncogenes, and impairing DNA repair mechanisms [143]. These modifications have the potential to disrupt finely tuned cell division processes in healthy cells via various molecular pathways, resulting in uncontrolled proliferation, genomic instability, metabolic reprogramming, and the acquisition of mesenchymal cellular characteristics, such as improved survival and the ability to migrate to sites of distant tissues [143].

Currently, three primary systems are recognized as the key drivers of epigenetic alterations: DNA methylation, histone modification, and expression or silencing of noncoding RNA (ncRNA) genes. These systems collectively initiate and sustain the dynamic landscape of epigenetic changes associated with cancer progression [144] [145,146]. Several cellular genes, encompassed in cell cycle regulation, apoptosis, DNA damage repair, cell adhesion, senescence, and survival, are altered in cancer cells, the result of the methylation patterns of their promoters, induced by the action of E6 and E7 oncoproteins [57]. The viral E6 and E7 oncoproteins work together to instigate hypermethylation of cellular genes [147]. E6 determines the degradation of p53 and the release of the transcription factor Sp1, which subsequently binds to the promoter of the DNMT1 gene, triggering its expression [147]. Likely, E7 forms a stable complex with pRB, releasing the transcription factor E2F, which then binds to the promoter of the DNMT1 gene, activating its expression [147]. These dual mechanisms converge to favor the production of DNA methyltransferase enzymes, thereby facilitating the hypermethylation of CpG islands and the subsequent silencing of critical cellular genes [145]. An example of this phenomenon involves the transcriptional silencing obtained through the hypermethylation of CpG islands within the promoter regions of the KIP1 and TP53 genes responsible for encoding p27 and p53 proteins, respectively. Both proteins play critical roles in cell cycle regulation. In the context of cervical cancer, this hypermethylation event is significantly more prevalent than in normal tissue [148]. A parallel process takes place with the hypermethylation of the promoter region of the homologous gene encoding phosphatase and tensin, a key player in the regulation of cell cycle progression. As a result, this tumor suppressor gene is silenced. Reduced expression of these genes is closely linked to increased cell proliferation and increased motility, a consequence of inactivation of the PI3-kinase-dependent signaling pathway [149]. 

Regulation of gene expression can also be modulated by histone modifications and nucleosome remodeling [150]. The addition of chemical groups to histone tails, including acetylation, methylation, phosphorylation, sumoylation, and ubiquitination, significantly affects the physical structure and transcriptional capacity of chromatin. These alterations in chromatin structure play a critical role in the regulation of essential cellular processes, including cell cycle control [151]. In particular, the reduction of histone acetylation levels is a critical factor in the development of cancer, as it leads to epigenetic silencing of tumor suppressor genes [152]. Key cellular genes central to this phenomenon include the tumor suppressor products, p53 and pRB [101]. They influence a wide range of genes involved in essential cellular processes [141,142]. When the HR-HPV E6 protein associates with the transcription factor Myc inside the cell, it triggers the activation of the hTERT gene promoter, achieved through the modulation of histone modifications via phosphorylation. This leads to high production of the telomerase enzyme within the infected cell, leading to cellular immortalization and, consequently, an increased risk of developing HPV-associated tumors [153,154,155]. An examination of tumor suppressor genes within urothelial carcinoma tissues, including RARβ2, E-cadherin, and β-catenin, has revealed a critical epigenetic alteration. Specifically, the promoters of these genes exhibit deacetylation, resulting in diminished or entirely absent gene expression. This impairment in gene expression significantly promotes the progression of tumor metastasis [156]. An assessment of HDAC3 expression in various cervical tissue samples, including normal tissue, moderate and severe cervical intraepithelial neoplasia (CIN), and urothelial carcinoma of the cervix, has yielded noteworthy findings. Notably, HDAC3 expression levels were markedly elevated in cancerous tissues when compared to normal tissue or cases of CIN2 and CIN3. This observation strongly implies that HDAC3 likely assumes a pivotal role in the progression of urothelial of the cervix carcinogenesis [122]. The E6 protein found in HR-HPVs has multifaceted effects, including the degradation of p53, the activation of telomerase, and the stimulation of various cellular oncogenes [157]. Recent research has uncovered a significant interaction between the E6 protein of HPV16 and the histone H3K4 demethylase KDM5C. This interaction results in the E6AP-mediated proteasomal degradation of KDM5C, dependent on E3 ligase activity. When examining CaSki cells, which are HPV16-positive tumor cells, it becomes apparent that they exhibit lower levels of KDM5C compared to tumor cells lacking HPV infection. This finding underscores the role of E6 in manipulating cellular processes by targeting KDM5C and provides insights into the molecular mechanisms underlying HPV-associated tumorigenesis. Reduced levels of KDM5C lead to increased expression of super-EGFR enhancers and c-MET oncogene [158]. 

The pathway of tumorigenesis, such as the development of HR-HPV-induced cervical cancer, can be significantly shaped by non-coding RNA (ncRNA)-mediated mechanisms. These can positively or negatively influence the expression of both cellular and viral genes, thus influencing the course of carcinogenesis [159]. These ncRNAs function as crucial regulators, driving tumor cell proliferation, mobility, and invasiveness, all while suppressing apoptosis and impairing cell adhesion [160]. Highly expressed long ncRNA 1 (CCHE1) has been identified as upregulated in HPV16-induced cervical carcinoma, with its expression levels being contingent on tumor size. Additionally, CCHE1 has been observed in other malignancies, including osteosarcoma, lung cancer, and hepatocellular carcinoma, as reported in various studies [161,162,163]. One of the extensively studied ncRNAs in the context of cervical cancer is HOTAIR. This lncRNA exerts control over gene expression by engaging with chromatin remodeling complexes, as indicated in the literature [164]. Interestingly, studies have unveiled that the HPV16 E7 protein can bind to HOTAIR, thereby disrupting its interaction with the polycomb repressive complex 2 (PRC2). These findings have been reported in multiple studies [165].

### 3.3. Epigenetic Modulators for the Treatment of HPV-Induced Infections and Tumors

The prominent involvement of epigenetic mechanisms in HPV infection and the subsequent development of carcinogenesis, along with their adaptable and reversible nature, underscores their suitability as promising targets for therapeutic approaches in addressing HPV infections and related tumors [11]. Investigating the potential therapeutic applications of these compounds has considerable significance, especially considering the absence of treatment strategies aimed at combating HPV diseases [166]. 

#### 3.3.1. HPV and HDAC Inhibitor

The process of histone acetylation involves the attachment of an acetyl group to lysine residues located on the extended tails of histone proteins [167]. Typically, this modification is linked to the activation of transcriptional processes and is under the control of two groups of contrasting enzymes: histone acetyltransferases (HATs) and histone deacetylases (HDACs) [168]. HATs facilitate the attachment of an acetyl group to the ε-amino group of lysine with the use of acetyl-CoA as a coenzyme. This process effectively neutralizes the positive charge of lysine, thus weakening the interaction between histones and DNA, ultimately making genes more accessible [169]. On the other hand, HDACs are responsible for eliminating acetyl groups from lysine residues, restoring a positive charge to lysine, and promoting tighter binding between histones and DNA. This process can lead to gene silencing and a more condensed chromatin structure, inhibiting gene expression [165]. The FDA has granted approval to various HDAC inhibitors as viable therapies for potential use in cancer treatment [170]. Growing evidence supports the utility of HDAC inhibitors in addressing hematologic malignancies and solid tumors [171,172]. Instances of FDA-approved drugs with documented interference with HPV-induced carcinogenesis include suberoylanilide hydroxamic acid (SAHA), trichostatin A (TSA), entinostat (MS-275), valproic acid (VPA), belinostat (PXD101), and panobinostat [173,174]. 

SAHA and TSA, both FDA-approved pan-inhibitors of HDACs, share structural similarities and effectively target class I and II HDACs [175]. SAHA’s mechanism involves impeding tumor growth and inducing cell cycle arrest in phase G1 [176]. It is applied in the treatment of various conditions, including pancreatic cancer, pancreatic adenocarcinoma, breast cancer, and multiple myeloma (NCT00948688, NCT00574587, and NCT00857324). Conversely, TSA acts by inhibiting the cell cycle and promoting apoptosis and is employed as an anticancer agent for breast and prostate cancer [177]. Cervical cancer cells are highly dependent on the expression of HPV E6 and E7 oncoproteins. E6 contributes to its oncogenicity in part by facilitating the rapid degradation of p53 and PDZ family tumor suppressor proteins. On the other hand, the E7 oncoprotein functions partly, by co-opting HDAC1/2. Considering this, Lin et al. postulated that combined inhibition of proteasome function and HDAC activity could synergistically and specifically induce cell death in cervical cancer cells by disrupting E6 and E7 signaling pathways. Their data revealed that the combination of bortezomib (a proteasome inhibitor) with TSA or SAHA (HDAC inhibitors) demonstrated synergistic cytotoxicity in HPV-positive cervical cancer cell lines, while it had no such effect on HPV cell lines-negative. Furthermore, when these combinations were administered to HeLa xenografts, they significantly delayed tumor growth compared to using either agent alone [178]. Due to the high involvement of HDACs in the processes of viral replication and cellular transformation, Banerjee et al. examined the impact of SAHA on productive high-risk HPV-18 infection within organotypic cultures of primary human keratinocytes. Their investigation revealed that SAHA led to a substantial decrease in E6 and E7 activities, effectively halting the amplification of viral and host DNA and the response to infection-associated damage. As a result, exposure to SAHA ended the production of viral progeny, thus preventing viral transmission and inducing DNA damage within infected cells, ultimately triggering apoptosis [179]. 

In 2014, the FDA approved the HDAC inhibitor PXD101 for the treatment of peripheral T-cell lymphoma [180]. Ongoing studies are evaluating its single-dose administration in patients with recurrent or pathological malignancies (NCT01583777). In vitro studies conducted on HPV-18 using organotypic cultures of primary human keratinocytes demonstrated that PXD101 treatment led to the inhibition of viral replication and induced apoptosis in infected HPV cells [179]. 

Panobinostat is an orally administered HDAC inhibitor for the treatment of melanoma and myelofibrosis [181]. It has also been combined with idarubicin and cytarabine for acute myeloblastic leukemia (NCT00840346). Wasim et al. assessed the impact of panobinostat on two cervical cancer cell lines, HeLa and SiHa. They investigated cell viability, apoptosis, oxidative stress, and mitochondrial function in response to HDAC inhibitor treatment. The findings reveal that panobinostat diminishes the viability of cervical cancer cells in a dose- and time-dependent manner, leading to cell cycle arrest in the G0/G1 phase in HeLa cells and in the G2/M phase in SiHa cells. It was proven that panobinostat exerts its apoptotic effect by enhancing the production of reactive oxygen species (ROS) and disrupting the mitochondrial membrane potential. It reduces the expression of the anti-apoptotic gene Bcl-xL while elevating the levels of the CDK inhibitor p21 and caspase-9. Moreover, panobinostat also augments histone H3 acetylation, indicating its inhibition of HDAC activity. This study highlights that panobinostat demonstrates a synergistic effect when combined with topoisomerase inhibitors, leading to heightened activation of caspase-3/7 activity compared to cells treated with panobinostat alone [182]. The impact of panobinostat on HPV-induced infection and carcinogenesis was also demonstrated by Banerjee et al. Their results reported panobinostat’s ability to directly interfere with the viral cycle, blocking genome replication and inducing tumor cell apoptosis [179]. 

VPA is a class I and II HDAC inhibitor approved for the treatment of breast cancer, myelodysplastic syndrome, acute myeloid leukemia, and spinal muscular atrophy in patients (NCT01900730, NCT00374075, NCT00326170). Faghihloo et al. examined the impact of VPA treatment on the expression of E-cadherin and E7. The results revealed that VPA treatment led to a more significant increase in E-cadherin levels in HPV-positive cell lines (HeLa and TC-1) compared to HPV-negative cell lines (MKN45 and HCT116). Additionally, the expression of the E7 oncoprotein decreased by a factor of 4.6 in infected cells [183]. In a study conducted by Coronel et al., a group of cervical cancer patients underwent combination therapy to improve treatment. Hydrolazine, a vasodilator, and VPA were added to standard therapy with cisplatin-topotecan. This therapeutic modification was associated with improved survival outcomes compared to patients who received cisplatin-topotecan monotherapy [184]. Subsequent phase III clinical trials involving patients with advanced cervical cancer revealed that those treated with the combination of chemotherapy, valproic acid, and hydralazine, experienced a longer median survival period (10 months) than those who received chemotherapy with placebo (6 months) [184]. 

MS-275 is a benzamide-based HDAC inhibitor belonging to classes I and IV, primarily prescribed for breast cancer patients [27]. Marques et al. investigated the impact of MS-275 on oral squamous cell carcinoma. The administration of this inhibitor resulted in a notable decrease in cell proliferation, followed by cell cycle arrest in the G0/G1 phase, and substantial induction of tumor apoptosis. This treatment also led to an elevation in ROS production and a significant reduction in cancer stem cells (CSCs). Furthermore, data indicated that MS-275 led to increased acetylation of histone H3 and histone H4, along with alterations in the expression of cell cycle-associated proteins, such as p21 [185]. A Phase I/II study is currently evaluating the combination of Bintrafusp Alfa (M7824), MS-275, and NHS-IL12 (M9241) to treat patients with HPV-associated cancer (NCT04708470). FDA-approved HDAC inhibitor and their respective clinical trial phases are summarized in Table 2. 

#### 3.3.2. HPV and DNMT Modulators

DNA methylation involves the addition of a methyl group (–CH3) to the fifth carbon position of cytosine within CpG dinucleotides, converting it into 5-methylcytosine [186]. This modification is closely associated with gene silencing and is catalyzed by DNA methyltransferases (DNMTs) using the coenzyme S-adenosyl-methionine (SAM) as a methyl group donor [187]. DNMTs can be categorized into two main classes, namely DNMT1 and DNMT3 [188]. DNMT1 is primarily responsible for preserving the methylation patterns on newly synthesized DNA strands during each replication cycle. In the absence of DNMT1, the replication machinery generates unmethylated DNA strands, leading to a gradual “dilution” of methylation and, consequently, a “passive” demethylation process, which doesn’t involve the active removal of methyl groups from existing methylated cytosines [189,190,191]. On the other hand, DNMT3a and DNMT3b are the methyltransferases predominantly involved in de novo methylation. Additionally, DNMT3L is a protein that lacks catalytic activity but is believed to play a regulatory role in de novo methylation by enhancing the binding of DNMT3 enzymes to DNA and stimulating their activity [192]. To date, several pieces of evidence have established connections between carcinogenesis and DNA hypermethylation [193,194,195]. Consequently, it is of fundamental importance to identify anticancer drugs capable of inhibiting DNMT. 

The FDA has approved 5-azacytidine and decitabine as drugs used to target DNMTs in the treatment of myelodysplastic syndrome and acute myeloid leukemia. In both mouse models and clinical investigations involving individuals with HPV-induced head and neck squamous cell carcinoma (NCT02178072), the use of 5-azacytidine has demonstrated promising results. It has been shown to reduce cell proliferation, suppress viral gene expression, and trigger apoptosis in a p53-dependent manner, as documented by Biktasova et al. [196]. Furthermore, in another clinical study involving patients with HPV-positive anogenital carcinomas and squamous cell carcinomas of the head and neck who are at high risk of recurrence after radiotherapy (NCT04252248), the administration of intravenous decitabine. However, the conclusive results of this study are pending. 

Of notable interest is a proposed clinical investigation aimed at evaluating the impact of orally administered decitabine in combination with durvalumab for patients with recurrent or metastatic carcinomas affecting the oral cavity, oropharynx, hypopharynx, or larynx. This study will also involve a comparative analysis of treatment response between HPV-positive and HPV-negative patients (NCT03019003) [197] (Table 3).

#### 3.3.3. Considerations: Epigenetic Drugs

The reversibility of epigenetic alterations introduces an interesting aspect of cancer treatment. Epigenetic therapeutic modalities show enormous promise in addressing a wide range of cancers. The examination of epigenetic mechanisms as possible therapeutic targets for HPV infections and associated cancers arouses considerable interest within the scientific community. However, the precise modulatory impact of epigenetic modifications on HPV infection and carcinogenesis remains enigmatic, requiring a deeper understanding of these intricate processes. The discovery of such HPV-induced mechanisms and the identification of novel viral noncoding RNAs could serve as potential sources for the development of triage biomarkers and promising pharmacological agents. Despite rapid progress in the field of epigenetics, significant gaps persist. In many cases, currently available therapies do not have precise specificity. Drugs targeting DNMTs and HDACs exhibit significant cytotoxicity due to their induction of global epigenetic alterations, resulting in undesirable consequences. Consequently, a more comprehensive analysis is crucial to identify domains unique to the epigenetic mechanisms underlying HPV infection and carcinogenesis. A better understanding of such mechanisms and the protein complexes that govern them will contribute to the refinement of therapeutic targeting, generating so-called smart drugs that will target epigenetic modifications limited to HPV disease. 

## 4. HPV: Genetic and Immunotherapy

### 4.1. Engineered T-Cell Therapy

Genetic modification of immune cells emerges as a highly promising strategy to augment innate anti-tumor responses of the immune system [198]. This approach gives immune cells greater anti-tumor specificity, allowing them to selectively target and eliminate tumor cells while sparing healthy tissue [199]. The incorporation of chimeric antigen receptors (CARs) into immune cells facilitates precise recognition and binding of antigens, which are often expressed by tumor cells [200]. 

Originally developed in T-cells (CAR-T), this technology has been extended to various types of immune cells, including natural killer (NK) cells and macrophages [201]. The fundamental structure of CARs comprises a single-chain variable fragment (scFv) derived from an extracellular antibody linked via a transmembrane domain to an intracellular CD3ζ signaling domain [202]. In subsequent years, the CD3ζ signaling domain has been associated with costimulatory domains, particularly CD28 or 4-1BB, which have become prevalent in commercially approved CAR-T cell therapies [203]. CD28 amplifies cytotoxic activity, while 4-1BB has been linked to an increased in vivo resistance of CAR-T cells [204]. Activation of these CAR components triggers downstream signaling pathways, ultimately resulting in potent cytotoxic effects against the target cell [205]. The success of CAR-T therapy hinges upon the capability of T-cells to discern and eradicate tumor cells, a process that necessitates the activation of T-cells and the subsequent secretion of pivotal cytokines, notably gamma interferon (IFN-γ) [206]. CAR-T has demonstrated remarkable efficacy in hematological malignancies, boasting response rates ranging from 70% to 90% [207]. However, when it comes to applying such therapy to solid tumors, formidable challenges arise. These challenges are primarily attributed to the immunosuppressive nature of the tumor microenvironment and the paucity of tumor-specific antigens that can be targeted [208]. As a result of the above-mentioned constraints, clinical investigations of CAR-based cell therapy for cervical cancer remain relatively limited (Table 4) [209]. Currently, only a few clinical studies have been dedicated to this specific application. Two major Phase I clinical trials are underway, one evaluating CAR-T-cells designed to target CD22 (NCT04556669) and another targeting CD70 (NCT05518253). Additionally, another study (NCT03356795) exploring the potential of CAR-T therapy against cervical cancers through targeting multiple antigens, including GD2, PSMA, MUC1, and mesothelin, is underway. Currently, this study has not published any results. Additionally, a phase I/II clinical trial (NCT01583686) previously evaluated CAR T-cell therapy directed against mesothelin. The clinical results of this study were somewhat less encouraging, with only one in fifteen patients achieving stable disease. A key limitation of CAR therapy lies in its reliance on targeting antigens that are visibly displayed on the surface of tumor cells [210]. As a result, this approach is unable to address intracellular proteins, which constitute a substantial portion of cellular proteins. In contrast, TCRs have the unique ability to recognize peptide fragments derived from intracellular proteins, although they are constrained by their dependence on MHC-mediated presentation [211]. These engineered TCRs consist of α and β chains, sourced from T-cell clones meticulously chosen for their robust target affinity. This designed ligand binding site is closely linked to native TCR signaling components, including the CD3δ, CD3ε, CD3γ, and CD3ζ domains [212]. Given their greater number of subunits and co-receptors, TCR-engineered T-cells have been observed to engage in more sustained immune synapses with the target antigen compared to CAR-T cells, increasing their potential for therapeutic efficacy prolonged [213]. 

Recent trials have consolidated TCR-T cell therapy as an innovative model to combat epithelial tumors, showing response rates ranging from 50% to 80% [214]. In the context of HR-HPV-induced cervical cancers, viral proteins emerge as attractive targets for TCR-based therapies due to their conspicuous absence in healthy cells [215]. HPV E6 and E7 oncoproteins present themselves as particularly interesting candidates for targeting, given their high expression levels in cervical cancer and robust evolutionary conservation [216]. Preclinical investigations yielded promising results, where engineered E7 TCR-T cells effectively facilitated the regression of HPV-positive tumors in mouse models. These findings highlight the potential of TCR-based therapies against these viral oncoproteins [217]. Furthermore, ongoing clinical trials are actively evaluating the therapeutic potential of engineered TCR-T cells directed against the viral oncoproteins E6 (NCT03578406) and E7 (NCT02858310), as detailed in Table 5. In the case of the latter, a recent update reported encouraging results, with 6 out of 12 patients enrolled demonstrating a partial response, while 4 patients achieved stable disease. Similarly, in another phase I/II clinical trial (NCT02280811) focused on evaluating engineered E6 TCR-T cells, 2 of 12 patients showed a partial response, and 4 of 12 patients maintained stable disease. These findings demonstrate the potential of TCR-based therapies in addressing the unique challenges posed by HR-HPV-induced cervical cancers. In a study conducted by Poorebrahim et al., the functionality of immune effector cells underwent significant enhancement through the strategic combination of the CAR (chimeric antigen receptor) approach with a TCR (T-cell receptor). In this investigation, they employed a clinically validated TCR designed specifically for targeting HPV16 E7 (E7-TCR) in conjunction with a newly engineered CAR designed to recognize the trophoblast cell surface antigen 2 (TROP2). This CAR incorporated intracellular costimulatory domains, namely CD28 and 4-1BB, although it did not include the CD3ζ domain. Flow cytometry analyses conducted as part of the study unveiled a remarkable upregulation of activation markers and the release of cytolytic molecules by NK-92 cells that had been genetically modified to express CD3, CD8, and both E7-TCR and TROP2-CAR. This heightened response occurred following co-incubation with HPV16-positive cervical cancer cells. Furthermore, the study demonstrated that E7-TCR/TROP2-CAR NK-92 cells exhibited superior antigen-specific activation and more potent cytotoxicity against tumor cells when compared to NK-92 cells expressing E7-TCR alone. These findings underscore the synergistic potential of co-stimulatory TROP2-CAR in collaboration with E7-TCR within NK cells, resulting in heightened signaling strength and enhanced antigen-specific cytotoxicity, which holds significant promise in the context of cancer immunotherapy [218].

#### Considerations: Immunotherapy

Antigen receptor-engineered T-cell therapy represents an emerging strategy for the treatment of cancer. CAR-T cells have demonstrated significant efficacy in the context of leukemia and lymphoma. Meanwhile, TCR-T cells have shown promising responses in selected soft tissue tumors when administered together with other anticancer agents. Although engineered T-cell therapy has promising potential for the treatment of HPV, there are notable knowledge gaps and areas of conflict within this innovative approach. The interaction between engineered T-cells and HPV-infected cells, including the dynamics of T-cell persistence and the long-term impact on the patient’s immune system, remains insufficiently understood. Controversies also target the optimal design of engineered T-cells to target HPV. Determining the most effective antigens to target, ensuring specificity to HPV-infected cells without causing unwanted side effects, and addressing potential resistance mechanisms are active areas of research and debate. The long-term safety of engineered T-cell therapy for HPV is also a key concern. Unraveling the potential for off-target effects and understanding the broader implications on overall patient health requires further exploration. Briefly, although engineered T-cell therapy for HPV is promising, ongoing research is essential to fill existing knowledge gaps and resolve controversies surrounding its implementation as a viable and safe treatment option.

### 4.2. CRISPR-CAS9

In recent times, CRISPR/Cas9 technology has emerged as a highly promising tool for inhibiting the expression of specific genes [219]. In the context of cervical cancer, viral oncogenes E6 and E7 play a critical role in the transformation of healthy cervical cells into cancerous cells. Notably, these oncogenes show unique expression in cancer cells, making them prime candidates for CRISPR/Cas9-based therapeutic interventions [80]. This innovative approach allows for the precise introduction of targeted mutations into the viral genome, effectively disrupting the virus’s ability to replicate, spread, and induce cellular transformation. Consequently, CRISPR/Cas9-based therapies have substantial potential to halt the progression of cervical cancer by disabling these crucial oncogenes [220]. 

In a study conducted by Zhen et al., CRISPR/Cas9 technology was exploited to precisely target the E6/E7 promoter region of HPV 16, thereby disrupting the expression of oncogenes in the HPV16-positive cervical cell line (SiHa). The result of this intervention led to a notable accumulation of p53 and p21 proteins, resulting in a significant reduction in the proliferative capacity of cervical cancer cells when evaluated in vitro. Subsequently, these modified cells were implanted subcutaneously into nude mice, demonstrating effective suppression of tumorigenesis and limited growth in the transplanted tumors. Furthermore, the survival time of mice carrying these tumors was significantly extended, underscoring the potential of CRISPR/Cas9 technology as a promising tool in the fight against cervical cancer [221]. 

In a separate investigation, researchers exploited HPV-18-positive cervical cancer cell lines by engineering them to constitutively express Cas9, combined with an adeno-associated virus (AAV) vector carrying a single, specifically targeted guide RNA (sgRNA) to the E6 gene (AAV -sgE6). This study involved transfection of the Cas9 gene into three HR-HPV-positive cervical cancer cell lines, HeLa, HCS-2, and SKG-I, to establish cell lines that consistently expressed Cas9. Using these engineered cell lines, the study meticulously examined genetic mutations and their frequencies, protein expression levels, apoptosis rates, and cell proliferation in vitro. Furthermore, the impact of AAV-sgE6 was evaluated in an in vivo cervical cancer mouse model by administering AAV-sgE6 directly into subcutaneous tumors. The results of this research revealed multiple mutations in the E6 genomic sequence of cervical cancer cells transduced with AAV-sgE6. Notably, these cells, after transduction with AAV-sgE6, showed reduced E6 expression, increased p53 expression, higher rates of apoptosis, and concentration-dependent growth suppression. Furthermore, when AAV-sgE6 was administered intratumorally, it significantly inhibited subcutaneous tumor growth in vivo, and, importantly, no adverse events attributable to AAV-sgE6 administration were observed [222].

Jubair et al. used a cervical cancer model system characterized by the persistent expression of E6 and E7 proteins. Their study demonstrated that when CRISPR/Cas9 was administered systematically in vivo using PEGylated liposomes, it produced the remarkable result of complete tumor eradication and subsequent survival of the treated animals. They conducted a comparative analysis to evaluate the effectiveness of the treatment and the impact of different Cas9 variants, namely wild-type (WT) Cas9 and highly specific FokI-dCas9. Their investigation revealed that the latter, FokI-dCas9, did not produce effective results. Furthermore, they delved into the field of high-fidelity repair mechanisms, finding that the repair process was relatively inefficient, occurring in only 6% to 8% of cells. In contrast, nonhomologous end joining (NHEJ) was highly efficient, occurring in approximately 80% of cells. Furthermore, the researchers explored the events that occur following gene editing within the tumors, clarifying that cell death was induced through apoptosis [223]. Moreover, Ehrke-Schulz et al. employed high-capacity adenoviral vectors (HCAdV) designed to express the complete CRISPR/Cas9 machinery, which was tailored specifically for HPV18-E6 or HPV16-E6. They conducted experiments using SiHa and CaSki cervical cancer cell lines, which contained HPV16 and HeLa cells harboring HPV18 genomes that were integrated into their cellular DNA. Additionally, HPV-negative tumor cells were included in the study as control. Following the administration of HPV-type-specific CRISPR-HCAdV, there was a discernible impact on the cervical cancer cells harboring HPV. Specifically, the expression of HPV type-specific CRISPR/Cas9 resulted in a marked reduction in the viability of HPV-positive cervical cancer cell lines, while HPV-negative cells remained unaffected. The transduced cervical cancer cells exhibited an elevated induction of apoptosis and reduced proliferation compared to untreated cells or HPV-negative control cells [224]. 

In approximately 8% of cervical cancers and up to 30% of oropharyngeal cancers, tumor cells harbor HPV DNA but do not express expression of HPV E6/E7 oncogenes. These specific tumors are referred to as “inactive” to distinguish them from “active” tumors, which are characterized by the presence of both HPV DNA and E6/E7 mRNA. Specifically, primary oropharyngeal cancers that are HPV-positive tend to show an active HPV status, whereas HPV-positive recurrent or metastatic cancers tend to show an inactive HPV status. It is worth mentioning that HPV-inactive cervical cancers often have mutations in the p53 [225].

In this context, Abboodi et al. undertook research to ascertain whether the loss of p53 could contribute to the development of HPV-inactive tumors [226]. Using CRISPR-Cas9 technology, they successfully knocked out the p53 gene (p53–KO), resulting in a remarkable five-fold reduction in E7 mRNA expression in immortalized human HPV16 differentiation-resistant (HKc/DR) keratinocytes. Interestingly, the study found that E7 expression was recovered in p53-KO cell lines in response to 5-Aza-2 deoxycytidine treatment, highlighting the potential role of DNA methylation in this process. Further analysis through in situ hybridization revealed that the p53-KO cell lines comprised mixed populations of E6/E7-positive and -negative cells. This indicates that the loss of p53 predisposes HPV16-transformed cells to become less dependent on the continued expression of HPV oncogenes for their proliferation, shedding light on the complex interplay between p53 and HPV in tumorigenesis. This evidence presents a comprehensive examination of CRISPR/Cas9 as a prospective therapeutic approach for cervical cancer induced by oncogenic HPV [226]. These studies underscore the urgency for novel therapeutic interventions in the realm of cervical cancer, signifying that CRISPR/Cas9 holds substantial promise as an exceptionally effective and precisely targeted therapeutic avenue. 

#### Considerations: CRISPR-CAS9

Using CRISPR-Cas9 technology to treat HPV introduces a cutting-edge approach with the potential for transformative results. However, this innovative strategy is not without unresolved knowledge gaps and areas of controversy. A significant challenge lies in the precision and specificity of CRISPR-Cas9 editing. Ensuring accurate targeting of HPV-infected cells while avoiding inadvertent changes to healthy cells remains an obstacle. Off-target effects, potential mutations, and broader impact on the host genome require thorough investigation to establish the safety and reliability of CRISPR-Cas9 in treating HPV. Another unresolved aspect concerns the long-term consequences of CRISPR-Cas9 treatment for HPV. Understanding the persistence of modified cells, the durability of therapeutic effects, and the potential for unexpected outcomes over a prolonged period is essential to ensure the long-lasting success and safety of this approach. Ethical considerations also contribute to the ongoing debate over CRISPR-Cas9 applications. Discussions about the ethical implications of human genome editing highlight the need for a thoughtful and responsible approach to integrating this technology into medical practices. Although the CRISPR-Cas9 treatment for HPV holds great promise, addressing these knowledge gaps and addressing the associated controversies are crucial steps to realizing its full potential as a safe and effective treatment option.

## 5. Conclusions and Future Directions

HR-HPVs are associated with approximately 5% of human cancers. Although highly effective prophylactic vaccines appear promising for preventing a large fraction of HPV-associated cancers, they do not protect against pre-existing infections or prevent malignant progression and are not expected to impact the frequency of these cancers. Meanwhile, millions of people will develop HPV-associated cancers, and many will die from these cancers around the world. It is critical to identify efficient treatments to control and, ideally, eradicate HPV-associated cancers. 

Numerous epigenetic alterations occurring in both the HPV and cellular genome have been identified, including DNA hypomethylation, hypermethylation of tumor suppressor genes, histone modifications, and ncRNA alterations. The discovery of HPV-induced epigenetic alterations and novel viral noncoding RNAs and methylation signatures opens possibilities for their application as targets for repurposed drug selection and design of novel drugs capable of modifying the epigenetic landscape to improve patient survival and quality of life based on comprehensive studies. 

The growing evidence highlights the growing potential for clinical applications of immunotherapeutic strategies involving CAR-T and TCR-modified T-cells in cancer management. TCR-T therapy has attracted considerable interest in the treatment of HPV-induced cancer. While CAR-T therapy may initially appear limited in scope, its exceptional results in hematologic malignancies continue to position it as a promising avenue in the field of solid tumors, including cervical cancer. In the context of HR-HPV-induced cervical cancers, for TCR therapy, viral proteins emerge as efficient targets for cell therapies, as they are not expressed in healthy cells. The E6/E7 oncoproteins are most targeted by the scientific community as they participate in the front line of the carcinogenesis process. 

CRISPR/Cas9 strategy has broadened the spectrum of potential therapeutic strategies to address HPV infections. Precise targeting of HPV oncoproteins holds promise for eradicating the viral reservoir and preventing cancer recurrence and advancement. This innovative approach allows for the precise introduction of targeted mutations into the viral genome, effectively disrupting the virus’s ability to replicate, spread, and induce cellular transformation. Consequently, CRISPR/Cas9-based therapies have substantial potential to halt the progression of cervical cancer by disabling these crucial oncogenes. 

Presently, a multitude of clinical and preclinical studies, grounded in diverse models and employing various treatment strategies, are systematically delving into pertinent matters. Their objectives encompass bolstering treatment efficacy, mitigating adverse reactions, and investigating the potential of synergistic application in conjunction with other therapeutic modalities. Epigenetic and genetic therapy holds a promising outlook for cervical cancer treatment.

## Figures and Tables

**Table 1:** Functions of HPV proteins.

Protein	Function	Source
E1	❖ It possesses ATP-dependent helicase activity, which is required for DNA recognition and synthesis.	[28]
E2	❖ It stabilizes the link between E1 and the origin of replication. ❖ It recruits cellular factors and enzymes necessary for DNA synthesis. ❖ It induces the transcription of E6 and E7 oncogenes. ❖ It directly induces apoptosis by binding the anaphase-promoting complex (APC) and p53. ❖ It inhibited the integration of the viral genome into the cellular one.	[30,31,32,34,65]
E4	❖ It induces cell cycle arrest in the G2 phase. ❖ It promotes viral replication through interaction with E2. ❖ It modifies the arrangement of cytokeratins in the cell cytoplasm, promoting the release of nascent virions. ❖ It induces transcription of the late L1 and L2 genes.	[38,39,66]
E5	❖ It increases cellular proliferative activity through interactions with EGF receptors. ❖ It allows evasion of the immune response, allowing the sequestration of MHCI in the Golgi apparatus and reducing the recognition of the virus by CD8 T-cells.	[42,43]
E6	❖ It promotes p53 degradation via the ubiquitin-dependent proteolytic pathway, allowing for uncontrolled cell proliferation.	[43,47]
E7	❖ It binds unphosphorylated factor Rb, resulting in the unprogrammed re-entry of cells into S-phase.	[56,57]
L1	❖ It is the major HPV structural proteins that form viral capsids.	[58]
L2	❖ It is the minor HPV structural proteins that form viral capsids.	[64]

**Table 2:** Summary of FDA-approved HDAC inhibitor involved against cancer therapy.

Epigenetic Drug	FDA Approved	Clinical Trial Phase	NCT Number	Deseas
SAHA	✓	I, II	NCT00948688 NCT00857324 NCT00574587	Pancreatic cancer Multiple myeloma Breast cancer
TSA	✓	I	NCT03828926	Relapsed or refractory Hematologic malignancies
VPA	✓	II, I, II	NCT01900730 NCT00374075 NCT00326170	Breast cancer Spinal muscular atrophy Myelodysplastic syndrome Acute myelogenous leukemia
PXD101	✓	I	NCT01583777 NCT00421889	Advanced cancer Ovarian cancer
Panobinostat	✓	I, II, III	NCT00840346 NCT01023308	Acute myeloblastic leukemia Multiple myeloma
MS-275	✓	I, II, II	NCT04708470 NCT00828854 NCT04708470	Cancer, solid tumor, microsatellite stable colon Cancer (MSS); ER + breast cancer Metastatic checkpoint Refractory HPV associated malignancies

**Table 3:** Summary of FDA-approved DNMT inhibitor involved against cancer therapy.

Epigenetic Drug	FDA Approved	Clinical Trial Phase	NCT Number	Deseas
5-azacytidine	✓	I, I, II	NCT02940483 NCT00886457 NCT00744757	Brain tumor recurrent cancer Myelodysplastic syndrome
decitabine	✓	I	NCT04252248	Head and neck cancer Anogenital cancer

**Table 4:** Clinical trials based on CAR-T against cervical cancer.

Trial N.	Target	Condition	Phase	Patients N.	Start	Status
NCT01583686	Mesothelin	Metastatic/unresectable Mesothelin-positive cancer	I, II	15	2012	Term. (2018)
NCT03356795	GD2, PSMA, MUC1, Mesothelin	Patients with stage III, IV, or relapsed cervical cancer	I, II	20	2017	Pending
NCT04556669	CD22 + PD-L1	Advanced malignant solid tumors	I	30	2020	Pending Est. compl.: 2025
NCT05518253	CD70	CD70-positive advanced/metastatic solid tumors	I	36	2022	Pending Est. compl.: 2025
NCT05468190	CD70	Advanced/metastatic CD70-positive solid tumors	I	48	2022	Pending Est. compl.: 2025

**Table 5:** Clinical trials based on TCR-T against cervical cancer.

Trial N.	Target	Condition	Phase	Patients N.	Start	Status
NCT02280811	E6	HLA-A*02:01-positive HPV-16-associated metastatic/recurrent cancer	I, II	12	2014	Compl. (2016)
NCT02153905	MAGE-A3	HLA-A 01-positive metastatic/recurrent cancer expressing MAGE-A3	I, II	3	2014	Compl. (2018)
NCT02111850	MAGE-A3	(HLA)-DP0401/0402-positive metastatic/recurrent cancer expressing MAGE-A3-DP4	I, II	21	2014	Compl. (2021)
NCT02858310	E7	HLA-A*02:01-positive HPV-16-associated metastatic/recurrent cancer	I, II	180	2017	Pending Est. compl.: 2026
NCT03578406	E6 + PD-1	Metastatic/recurrent HPV-16-positive cancers	I	20	2018	Pending
NCT05357027	E6	HLA-A*02- and HPV16-positive metastatic/recurrent positive cervical cancer	I, II	18	2022	Pending Est. compl.: 2024
NCT05122221	E7	HLA-A*02- and HPV16-positive cancer	I	12	2022	Pending Est. compl.: 2024

## Data Availability

Data is contained within the article.
